# Reliability and validity of 3 different Chinese versions of the Oxford knee score (OKS)

**DOI:** 10.1186/s42836-020-00049-1

**Published:** 2020-10-21

**Authors:** James Reeves Mbori Ngwayi, Jie Tan, Ning Liang, Daniel Edward Porter

**Affiliations:** 1 School of Clinical Medicine, Tsinghua University, Beijing, 100084 China; 2 Tsinghua University Haidian district, Zijing Apartment 21, Beijing, 100084 China; 3 Department of Orthopedics, Beijing Huaxin Hospital, Clinical Medicine School, Tsinghua University, Beijing, 100016 China

**Keywords:** Patient reported outcome measures, Reliability, Knee osteoarthritis

## Abstract

**Background:**

Different Chinese versions of the Oxford Knee Score (OKS) are available for knee arthritis assessment. These include the Malaysian, Hong Kong and Singaporean Chinese versions with slight variations in wordings and use of Cantonese in the Hong Kong Version. This study evaluated the validity and reliability of the different Chinese OKS versions in Mainland China.

**Methods:**

One hundred ninety four China mainland-based patients participated in the study, each being diagnosed with knee osteoarthritis. The patients were randomly assigned into 3 groups: Group A completed the Malaysian OKS; Group B completed the Singaporean OKS; Group C completed the Hong Kong OKS. Participants also completed the 36-item Short Form Survey (SF 36). The electronic versions of the questionnaires completed by the patients were sent to smart devices via a social media platform.

**Results:**

Interclass coefficients for test-retest reliability of the groups were 0.917 in group A, 0.921 in group B, 0.824 in group C. Cronbach alpha results for internal consistency of the 3 groups were: 0.912 in group A, 0.896 in group B, 0.846 in group C. Spearson correlation results with individual SF-36 domains were as follows: Group A showed strong negative correlations with bodily pain and physical function domains; group B exhibited moderate negative correlations with the bodily pain and physical function domains; group C revealed strong negative correlations with the bodily pain and physical function domains and weak negative correlations with vitality domain.

**Conclusions:**

Different Chinese versions of the OKS showed good reliability and convergent validity in mainland samples of patients with knee osteoarthritis, supporting their use in research and other related studies.

## Introduction

The oxford knee score (OKS) is a commonly used patient-reported outcome measure originally designed specifically for the evaluation of joint replacement procedures [1] but its use has now extended to involve pharmacological treatment, physiotherapy and fractures [2]. OKS versions presently exist in many different languages, with 4 approved Chinese versions, including OKS Malaysian Chinese, OKS Hong Kong Cantonese and OKS Singapore Chinese versions, which were all validated in their respective regions [3, 4] and a Mainland Chinese OKS version [5]. We conducted a systematic online database search in PubMed, Embase, Web of Science, China Knowledge Resource Integrated Database (CNKI), WeiPu database (VIP) and WanFangData on the use of OKS scores in Mainland China, with time frame ranging from inception up to 2017. In addition to the aforementioned versions, this search revealed 3 more OKS Chinese versions in use. It is imperative to use high-quality validated questionnaires since poorly-designed questionnaires can potentially lead to unreliable and misleading results [6]. The English language OKS versions in use in different countries are very similar, but there are differences in expressions and use of words among various Chinese versions. Moreover, the OKS Hong Kong version uses traditional Cantonese characters. The aim of the study was to carry out validation and reliability studies of the 3 non-mainland Chinese versions to ascertain their interchangeable usage in mainland China.

## Materials and methods

The Oxford Knee Score (OKS) has 12-items, each having 5 ordinal response options over a recall period “during the last 4 weeks.” Each question is scored on a 0-to-4 point scale with 4 being the best outcome and the overall scores range from 0 to 48 [2].

Permission and licence for the use of OKS scores were granted by Oxford Innovation. Smart device-compatible electronic versions of all the scores were designed according to recommendations from Oxford Innovation and approved for use in the study. An electronic version of the SF-36 was also designed for patient completion.

WeChat® (Tencent, Shenzhen, China) application is a widely used social media platform in Mainland China, available over a wide variety of smart devices. The electronic versions were designed using online software tools which were compatible with the WeChat® platform. This study was approved by the Clinical Research Ethics Committee of our institution.

### Patients

One hundred ninety-four patients participated in the study, and were recruited over a period of 4 months from the General Orthopaedic Outpatient Clinic and Arthroplasty Specialists Clinic of our level-3 general hospital in Beijing. Participants were diagnosed with knee osteoarthritis on the basis of clinical symptoms and signs and the presence of positive radiographic findings as confirmed by two orthopaedic surgeons. The inclusion criteria were: (1) an ability to read and write Chinese, (2) having been diagnosed with knee osteoarthritis, (3) having access to and being able to use WeChat® platform. Participants were either newly diagnosed or had new onset of symptoms following a previous diagnosis. Patients on ongoing treatment were not included. Participants’ demographics, including gender, age and knee side involved, are listed in Table 1.

**Table 1:** Participants' demographics

Characteristics	Group A (*n* = 63)	Group B (*n* = 67)	Group C (*n* = 64)
Gender			
Female	37	29	39
Male	26	38	25
Knee Side			
Left	19	23	21
Right	20	23	22
Both	24	21	21
Mean (SD) Age (years)	48.16 (12.79)	51.58 (12.89)	47.27 (12.69)

*SD* Standard Deviation

Participants were randomly assigned into 3 groups. Group A (OKS Malaysia Chinese) had 63 patients, group B (OKS Singapore Chinese) consisted of 67 patients and group C (OKS Hong Kong Cantonese) was comprised of 64 patients. Sample sizes referred to recommendations from previous studies that at least 50 subjects should be included for comparison studies [7, 8] (Table 1).

The participants received the questionnaires via smart devices after consultation and were briefly instructed on how to complete the forms. During the form completion, the patients were asked to point out any difficulties in language comprehension of questions of the Score. During the process, questions were randomly selected and patients asked to explain the content. Participants were sent OKS questionnaires a second time via WeChat® platform for reliability analysis.

### Psychometrics

To asses test and retest reliability, patient responses of the first and second OKS measurements were compared. Previous studies used a time frame of 1–14 days between the completion of the scores, during which a patient's clinical status is unlikely to experience any major changes in the absence of specific intervention [1, 9]. Test-retest reliability was calculated using the intraclass correlation coefficient (ICC) to evaluate reproducibility. Cronbach’s α coefficient was used to measure internal consistency.

The construct validity was examined by means of convergent validity and divergent validity. The OKS scores were compared to the various domains of the SF-36 by calculating the Spearman’s rank correlation coefficients (ρ). The SF 36 assesses health under two headings: Physical Component Summary (PCS), which includes Physical Functioning (PF), Role Physical (RP), Bodily Pain (BP) and General Health (GH) and Mental Component Summary (MCS), which involves Vitality (VT), Social Functioning (SF), Role Emotional (RE) and Mental Health (MH). According to studies from Juniper et al, correlation values of > 0.50, 0.35 to 0.50, and < 0.35 can be interpreted as having strong, moderate, and weak correlation, respectively [10]. On the basis of information, we hypothesized there are strong correlations between OKS and related domain scores of the SF- 36 and weak correlations with non-related domains on the SF-36.

The statistical analysis was performed using SPSS® Version 20.0 software package (SPSS Inc., Chicago, IL, USA).

## Results

### Reliability studies

The OKS was completed a second time by 38 patients from Group A with an average time 3.26 (1.79) days from the first completion; 35 patients in Group B with an average time of 2.69 (1.07) days; 38 patients from Group C with an average time of 3.08 (1.17) days. The test-retest reliability calculated with ICC in all 3 OKS groups was 0.917, 0.921, and 0.824 respectively (Table 2). The Cronbach’s α coefficient for the 3 groups was 0.912, 0.896 and 0.846, respectively (Table 3).

**Table 2:** Test and retest reproducibility for 3 OKS versions

OKS Version	ICC	95% CI	*p* value
Group A	0.917	0.841–0.957	< 0.001
Group B	0.921	0.844–0.960	< 0.001
Group C	0.824	0.666–0.910	< 0.001

**Table 3:** Internal consistency for 3 different OKS versions

OKS version	Cronbach’s alpha
Group A	0.912
Group B	0.896
Group C	0.846

### Validity studies

Group A showed strong negative correlations with related domains of the SF-36, bodily pain (ρ = −0.724, ρ < 0.001) and physical functioning (ρ = −0.538, *p* < 0.001). Group A showed weak correlations with unrelated domains: vitality, role emotional and mental health but these correlations were not significant (ρ > 0.05). Group B exhibited moderate correlations with related domains of the SF-36, bodily pain (ρ = −0.495, *p* < 0.001), physical function (*p* = −0.406, *p* < 0.001). Group B bore weak correlations with unrelated domains: vitality, role emotional and mental health but these were not significant (*p* > 0.05). Group C reveled strong negative correlations with physical functioning (ρ = −0.655, *p* < 0.001), bodily pain (ρ = −0.565, *p* < 0.001, and weak negative correlations with unrelated domains vitality (ρ = −0.311, *p* < 0.05). Correlations with mental health and role emotional were not significant (Table 4).

**Table 4:** Spearman correlation between different OKS groups and SF-36

SF-36	Group A	Group B	Group C
PCS			
PF	−0.538^a^	−0.406^a^	−0.655^a^
RP	−0.479^a^	−0.198	−0.263
BP	−0.724^a^	−0.495^a^	−0.565^a^
GH	−0.325^b^	−0.123	−0.426^a^
MCS			
VT	−0.050	−0.107	−0.311^b^
SF	−0.328^b^	−0.438^a^	−0.353^b^
RE	−0.097	−0.101	−0.272
MH	0.189	−0.050	−0.286

^a^Correlation is significant at the 0.01 level (2-tailed), ^b^Correlation is significant at the 0.05 level (2-tailed), *MCS* Mental Component Summary, *PCS* Physical Component Summary

*PF* Physical Functioning, *RP* Role Physical, *BP* Bodily Pain, *GH* General Health, *MCS* Mental Component Summary, including *VT* Vitality, *SF* Social Functioning, *RE* Role Emotional and *MH* Mental Health

## Discussion

Currently, there are four different versions of the Chinese OKS, and all have been validated in sample populations in their various regions. Knee-related studies in mainland China using mainland-based samples employed various Chinese OKS versions. Beaton et al studied the cross-cultural adaptation guidelines and suggested that the same language questionnaires used in different countries have to undergo cross-cultural adaptation and psychometric analysis [11]. So, this study analyzed the psychometric properties of the available Chinese OKS versions in a mainland sample population so as to demonstrate their feasibility for use in Mainland-based studies.

Test-retest reliability calculation in the different groups showed excellent results, with ICC > 0.8 and accepted threshold ≥0.75 [12]. Internal consistency test in terms of Cronbach’s α yielded equally good results, with ICC > 0.80 and accepted threshold ≥0.7 [13]. Reliability results were similar to those obtained on the different OKS versions in their respective geographical settings, with all > 0.80.

In this study, construct validity was assessed by using only SF-36. Factor analysis of previous studies demonstrated that OKS could be composed of 2 factors, i.e., knee pain and knee dysfunction [9]. We thus expected strong correlations with similar related domains of the SF-36. The present study indicated a convergent validity. Results showed correlations between similar domains in the OKS, with moderate to strong correlations found with bodily pain and physical function, which was consistent with our hypothesis. Validation studies from the sample study in Singapore showed strong negative correlation with the Physical Functioning domain [4]. Strongest correlations in validation studies from the Hong Kong sample were with Physical Functioning and Bodily Pain domains [3]. The mainland Chinese version that used Pearson correlation coefficient for validation studies revealed moderate correlations with both the physical component summary of the SF-36 as well as the mental component summary [5].

The present Study utilized electronic from versions instead of the traditional paper-based formats, and the overall feedback from the participants was acceptable. Patient Reported Outcome Measures (PROMs) primarily have to be completed by the patients and ‘e-PROMs’ provide a relatively inexpensive and fast way of participants' follow-up, and are especially useful for patients seeking consultation from other cities, a common phenomenon in large hospitals in tier-1 cities. Previous studies have shown patients’ preference in the use e-PROMs, with their advantages including convenience and economy in terms of time, manpower and money. Errors due to manual transfer of paper data to electronic devices can also be avoided by using e-PROMs [14]. Results from meta-analysis on 65 studies conducted by Gwaltney CJ et al strongly suggested an equivalence between paper-based and e-PROMs [15]. The design of e-PROMs also has to follow strict guidelines, which stipulate that moderate and substantial modifications, including changing the wordings of the items or response options, should have evidence through equivalence studies and full psychometric testing. The e-PROMs used in this study involved only minor modifications and did not require psychometric studies prior to use [16]. However, patients brought up the issue of privacy and protection of information, which is a major security concern with smart devices. No personal or private information was collected and patients could provide their initials rather than full names. In administering e-PROMs and building databases, healthcare institutions need to scrupulously protect the systems to ensure the security of patient data.

## Conclusion

Different Chinese versions of the OKS were proven to be reliable and valid in mainland samples of patients with knee osteoarthritis, thereby supporting their use in research and other related studies.

## Data Availability

The datasets generated during the current study are not publicly available due to the fact that some data sets contain participants’ personal information, such as names and phone numbers.

## Abbreviations

OKS: Oxford Knee Score
SF 36: 36-Item Short Form Survey
CNKI: China Knowledge Resource Integrated Database
VIP: WeiPu database
ICC: Intraclass correlation coefficient
PROMs: Patient Reported Outcome Measures
